# The Many Faces of Human Leukocyte Antigen-G: Relevance to the Fate of Pregnancy

**DOI:** 10.1155/2014/591489

**Published:** 2014-03-04

**Authors:** Mette Dahl, Snezana Djurisic, Thomas Vauvert F. Hviid

**Affiliations:** Department of Clinical Biochemistry, Centre for Immune Regulation and Reproductive Immunology (CIRRI), Copenhagen University Hospital (Roskilde) and Roskilde Hospital, 7-13 Køgevej, 4000 Roskilde, Denmark

## Abstract

Pregnancy is an immunological paradox, where fetal antigens encoded by polymorphic genes inherited from the father do not provoke a maternal immune response. The fetus is not rejected as it would be theorized according to principles of tissue transplantation. A major contribution to fetal tolerance is the human leukocyte antigen (HLA)-G, a nonclassical HLA protein displaying limited polymorphism, restricted tissue distribution, and a unique alternative splice pattern. HLA-G is primarily expressed in placenta and plays multifaceted roles during pregnancy, both as a soluble and a membrane-bound molecule. Its immunomodulatory functions involve interactions with different immune cells and possibly regulation of cell migration during placental development. Recent findings include HLA-G contributions from the father and the fetus itself. Much effort has been put into clarifying the role of HLA-G during pregnancy and pregnancy complications, such as preeclampsia, recurrent spontaneous abortions, and subfertility or infertility. This review aims to clarify the multifunctional role of HLA-G in pregnancy-related disorders by focusing on genetic variation, differences in mRNA stability between *HLA-G * alleles, differences in HLA-G isoform expression, and possible differences in functional activity. Furthermore, we highlight important observations regarding *HLA-G * genetics and expression in preeclampsia that future research should address.

## 1. Introduction

The human leukocyte antigen (HLA)-G is a HLA class Ib protein, which in contrast to the highly polymorphic classical HLA molecules shows limited polymorphism and restricted tissue distribution and has a unique alternative splice pattern [1–3].

HLA-G is expressed as several different splice variants including four membrane-bound (HLA-G1 to -G4) and three soluble isoforms (HLA-G5 to -G7). In addition, both membrane-bound *β*2-microglobulin (*β*2m)-linked and free dimers, membrane bound *β*2m-free heavy chains, and possibly soluble *β*2m-free dimers have been reported [1, 4–6]. Approximately 50 *HLA-G* alleles corresponding to 16 HLA-G proteins have been reported (The IMGT database; Nov. 2013). Investigations of *HLA-G* genetics in relation to risk of certain pregnancy complications have increased during recent years [7–9].

The expression of HLA-G was first described in placenta during pregnancy as the conventional *β*2m-linked membrane-bound form, and subsequently several other isoforms have been reported in this compartment [1, 10]. To date, there is a good amount of evidence to support that the extra-villous trophoblast (EVT) cells express membrane-bound HLA-G1, soluble HLAG-5/-G6, and possibly other isoforms, whereas the HLA-G5 and HLA-G2/-G6 expression in villous trophoblast (VT) and syncytiotrophoblast (ST) cells have been proposed but are still a matter of controversy [6, 11, 12]. Furthermore, membrane-bound HLA-G1 can be shed from the membrane and released as soluble HLA-G1 [13]. In addition to placental expression, soluble HLA-G (sHLA-G) has been detected in peripheral blood from men, pregnant and nonpregnant women, follicular fluid, fertilized oocytes, and in male reproductive tissues including semen [14–18]. It is still not clear, however, exactly which of the isoforms of HLA-G that are responsible for the immunomodulatory functions during pregnancy.

HLA-G has been shown to be a ligand for the immune receptors immunoglobulin-like transcript 2 (ILT-2), ILT-4, and the killer immunoglobulin-like receptor 2 (KIR2DL4) [19–22]. The immunomodulatory effects that result from these interactions include inhibition of T cells, inhibition of NK cell proliferation and cytotoxic functions, enhancement of the generation of regulatory T cells, inhibition of the differentiation of antigen-presenting cells (APC), and alterations in cytokine secretion [23, 24].

During pregnancy, immunoregulatory functions must be initiated to secure acceptance of the semiallogenic fetus. The fetus displays antigens inherited from the father on the cell surfaces. Originally, the maternal acceptance of the fetus was explained partly by the fact that trophoblast cells lack expression of the highly polymorphic classical HLA-A and -B molecules and partly because the immune system shifted from a T helper 1 (Th1) cytokine profile towards a Th2 cytokine profile [33, 34]. However, this would generate a problem in relation to natural killer (NK) cell-mediated lysis of cells lacking HLA expression; uterine NK cells constitute a large part of the immune cells in the uterine compartment [35]. This dilemma was overcome, when the expression of nonpolymorphic HLA class Ib molecules, HLA-E, -F, and -G, on EVT cells, was discovered and explored. EVT cells also express HLA-C, apparently in low amount [36]. The restricted tissue distribution of HLA-G and its immunomodulatory functions have generated much effort into clarifying the function of HLA-G during pregnancy, and which role HLA-G might have in pregnancy complications, such as preeclampsia, recurrent spontaneous abortions, and subfertility or infertility.

Preeclampsia is a pregnancy disorder that can be roughly divided into two stages [37]. The first is characterized by the trophoblast migration into decidua creating the first materno-fetal interface, and the second is when the syncytiotrophoblast comes into direct contact with maternal blood. The disorder affects 2–7% of all pregnancies in varying degree from mild hypertension, proteinuria, and oedema to kidney and liver dysfunction, impairs the blood coagulation system, and in worst cases cerebral haemorrhage [8]. The pathology of this disorder is still unknown, but it has been proposed that preeclampsia evolves from dysfunctional immunoregulation. As a first step, during first trimester, it has been hypothesised that lack of an adequate immunological response may lead to failure of trophoblast invasion and failure of spiral arteries remodelling, resulting in poor blood supply and possibly hypoxia in the placenta. At the second step during second trimester, where signs of preeclampsia are recognizable in the maternal organ systems, it has been difficult to identify the triggering factors. Several studies have linked *HLA-G* genotypes and aberrant HLA-G protein expression to preeclampsia; however, other studies have not obsered any significant associations [38–41]. In addition, several studies have linked certain *HLA-G* genotypes and aberrant HLA-G protein expression to the risk of recurrent spontaneous abortions (RSA) [9].

This review aims to clarify the role of HLA-G in pregnancy-related disorders such as preeclampsia by focusing on genetic variation, differences in mRNA stability between different *HLA-G* alleles, differences in HLA-G isoform protein expression, and possible differences in receptor interactions and functional activity. The review will also highlight important and partly conflicting observations regarding *HLA-G* genetics and HLA-G expression in preeclampsia that further research needs to address.

## 2. *HLA-G* Polymorphisms in relation to Alternative Splicing and Protein Expression

**Figure 1:**
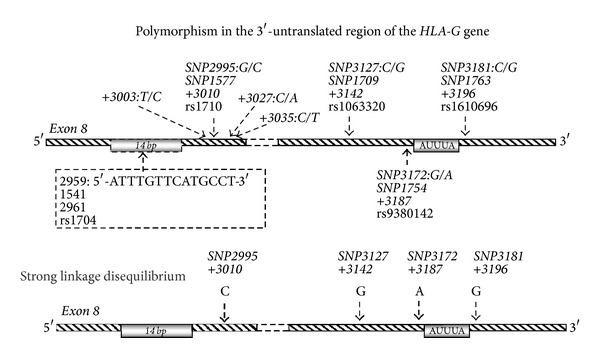
Polymorphisms in the 3′-untranslated region (3′UTR) of the *HLA-G* gene. The 3′UTR haplotype shown below has been associated with the risk of developing severe preeclampsia [25]. (Nomenclature used by different authors is shown for clarification. “*SNPxxxx*,” for example, *SNP3127*, is based on the original publication of the HLA-G gene sequence by Geraghty et al. [26] and the study of HLA-G 3′UTR haplotypes in cases of severe preeclampsia by Larsen et al. [25]; “*+xxxx*,” for example, *+3142*, by the publication by Castelli et al. [27]. (Based on [25, 27, 28])).

**Figure 2:**
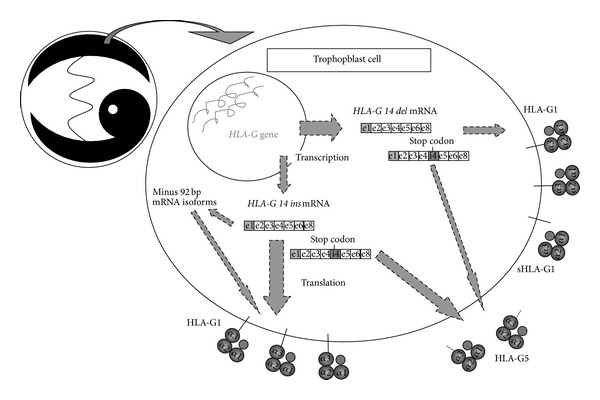
A simplified illustration of current status regarding the dynamic differences in transcription, mRNA stability, and translation in (extravillous) trophoblast cells between the *14 bp del* and *14 bp ins HLA-G* alleles. Only the full-length mRNA isoforms are shown for clarity. The relative thicknesses of the specific arrows are putative and should be interpreted with caution. The effect of the *HLA-G 14 bp ins/del* polymorphism in the 3′-untranslated region (3′UTR) on HLA-G expression may be influenced by linkage disequilibrium with single nucleotide polymorphisms (SNPs) in the 3′UTR, especially the *+3142* and *+3187* SNPs according to one study [29], and in the 5′-upstream regulatory region. (Based primarily on [1, 30–32]).

Given the immunoregulatory functions of HLA-G, studies have focused on possible gene variations influencing HLA-G expression. Harrison et al. reported a *14 bp insertion/deletion* (*14 ins/14 del*) polymorphism in the 3′-untranslated region (3′UTR) of the *HLA-G* gene at position +2961 (Figure 1) [42]. A summary of the most important known differences in posttranscriptional processing, protein expression, and functional activity of the *14 del HLA-G* allele and *14 ins HLA-G* allele is depicted in Table 1 and Figure 2.

In the current review, the positions of polymorphisms in the 3′UTR of the *HLA-G* gene are listed as in Castelli et al. [27]. These positions differ by 15 nucleotides after the *14 bp ins/del* polymorphism when compared to the original published *HLA-G *gene sequence by Geraghty et al. [26]. Nucleotide +1 is the adenine (A) of the first translated ATG. Furthermore, *HLA-G* allele nomenclature is in the current review listed according to the WHO nomenclature versions (The IMGT database) used in the specific original publications reviewed, because conversions to the current nomenclature can be difficult and inaccurate.

The *14 ins * allele has been associated with significantly lower *HLA-G* mRNA levels in first trimester trophoblast cells compared to the *14 del* allele [30, 41]. Furthermore, studies have shown unique alternative splice patterns in relation to the *14 ins * allele, where 92 bp are spliced out due to an introduction of a cryptic branchpoint not found in the *14 del* allele [1, 30]. This alternative splicing was more prominent in the *G∗010103 *(*G∗01:01:03:0x*) allele compared to the *G∗010102 *(*G∗01:01:02:0x*) allele, and interestingly, homozygous individuals carrying the *G∗010103 *allele showed the same HLA-G1 expression as the high secretor *G∗010101* (*G∗01:01:01:0x*) allele, and even higher expression of HLA-G2/-G4. Another study found that, when the 92 bp is deleted, a more stable *HLA-G* transcript is obtained [31]. This has led to a hypothesis that the deletion of 92 bp might be a compensatory mechanism in individuals carrying the *14 ins * allele in an attempt to increase sHLA-G protein expression.

In placental tissue, a reduced expression of HLA-G has been linked to preeclampsia, but these studies did not differentiate between membrane-bound HLA-G and sHLA-G; and most of these studies did not examine a possible association with the *14 bp* polymorphism [40, 41, 43, 44]. A number of studies have reported an association between fetuses carrying the *14 ins * allele and/or the *14 ins/14 ins * HLA-G genotype and risk of preeclampsia [25, 38, 41, 45, 46]. On the other hand, some studies have not observed any association [39].

Several studies have found that the *14 ins * allele is associated with decreased levels of soluble HLA-G in blood plasma [47–50], while a few studies found no association [51, 52]. In most of the studies, sHLA-G has been determined with a commercially available sHLA-G enzyme-linked immunosorbent assay (ELISA) kit (Exbio, Praha), based on the capture antibody MEMG/9, capturing sHLA-G1/-G5 in association with *β*2m and a detecting antibody against *β*2m. Interestingly, the study by Wu et al., who failed to report an association, used a different ELISA assay with a higher limit of detection, compared to the Exbio kit. This could account for the differences in results, whereas there is no obvious explanation to the reported lack of association in the study by Zhang et al. examining children with atopic asthma and positive controls. In a study by Rizzo et al., sHLA-G1 and HLA-G5 were determined by performing two different ELISA assays: one capturing both sHLA-G1 and HLA-G5 and one capturing only HLA-G5 by the use of the monoclonal antibody (mAb) 5A6G7, which is specific for HLA-G5/-G6 [53]. Surprisingly, this showed that women with severe preeclampsia had significantly higher levels of soluble HLA-G5 than women with uncomplicated pregnancies. In addition, there was a trend towards lower sHLA-G1 expression in women with severe preeclampsia [53]. This is in accordance with a study of *HLA-G *mRNA expression in preeclampsia, in which a high expression of *HLA-G5* mRNA was detected in preeclampsia compared to controls [54]. Also, it is partly in accordance with another study measuring HLA-G5 levels in blood plasma of pregnant women in relation to the *14 bp* polymorphism. This study showed no difference in HLA-G5 levels between pregnant women and control women, and levels of HLA-G5 did not seem to change during pregnancy. However, HLA-G5 levels were higher among women carrying the *14 ins * polymorphism either displaying heterozygosity or homozygosity than women without the *14 ins * allele [55].

In addition to the *HLA-G 14 bp* polymorphism, other regions of the *HLA-G* gene have been studied for possible association with HLA-G protein expression. The null allele *G∗01:05N* is defined by a single nucleotide deletion polymorphism in codon 130 (also known as *1597C*) in exon 3 leading to a frameshift mutation resulting in abnormal full-length HLA-G1 and -G5 isoforms. However, other alternatively spliced HLA-G isoforms lacking exon 3 are generated, and some studies support that these isoforms might also possess functional capacity resembling the function of the full length proteins [56]. This is further supported by recent experiments with synthetic HLA-G protein variants [57]. One study found that sHLA-G levels in healthy pregnant women were significantly lower in women carrying this *1597C* deletion mutation [58]. In addition, it was shown that the deletion mutation was more frequent in a group of preeclamptic women compared to normal controls.

Recently, increasing interest has been drawn towards examining single nucleotide polymorphisms (SNPs) in the 3′UTR region of the *HLA-G* gene. One study examined a C/G SNP at position +3142 in relation to microRNAs (miRNAs) [59]. MicroRNAs are noncoding single-stranded RNAs modulating gene expression by targeting mRNA. Based on thermodynamic calculations, the authors hypothesized that the binding of miRNA would be more stable in genotypes carrying a G at position +3142 compared to genotypes carrying a C. The sHLA-G level in JEG-3 cells expressing the *+3142G* SNP was markedly decreased after transfection with miRNA-148a. Although no comparison with the *3142C* genotype was made, this study indicates that *+3142G* might be associated with decreased expression of HLA-G [59]. Yie et al. found that a C/A SNP at position +3187 of exon 8 resulted in reduced half-life of mRNA-transcripts [60]. Interestingly, the *14 ins * allele is in linkage disequilibrium with both the *+3142G* and the *+3187A* SNPs. This illustrates that linkage disequilibrium in the *HLA-G* gene region plays a pivotal role. Other studies have identified more SNPs in the 3′UTR as well [27, 28, 61]. Larsen et al. studied *HLA-G* 3′UTR polymorphism in severe preeclamptic cases and controls in a North European population [25]. Castelli et al. showed that SNPs in the 3′UTR are target sites for different miRNAs [61]. In the Brazilian population, Castelli et al. tried to characterize polymorphisms and examine the linkage disequilibrium between them. Interestingly, they were able to group the gene variations into eight different UTR haplotypes [62]. *UTR-1* and *UTR-2* were the most frequently distributed, accounting for 52% of the haplotypes. On this basis, Di Cristofaro et al. examined the correlation of the UTR haplotypes with sHLA-G expression and found that *UTR-1* homozygous individuals displayed high secretion, whereas individuals homozygotic for the *UTR-2* haplotype were low secretors [63]. Furthermore, this study found that the highest secretors were the *UTR-5*, regardless of whether individuals were homozygous or heterozygous. In a recent study, however, *UTR-5* was found to be a low secretor [29]. A possible explanation to these contradictory results is that 5′URR polymorphisms affecting expression that are associated with the *UTR-5* haplotype may vary among populations. Furthermore, although the ELISA in the two studies was both based upon capture of sHLA-G1/HLA-G5 with the MEM-G/9 mAb, the study by Martelli-Palomino et al. used an in-house ELISA with a HLA-G5 protein as standard and tested blood *plasma* samples, while the study by Di Cristofaro et al. used the commercial Exbio sHLA-G kit with no well-defined standard and tested blood *serum* samples [29, 63]. Serum samples have been shown to be less reproducible in relation to sHLA-G measurements [64]; however, differences in sHLA-G concentrations may turn out more pronounced when analysing serum samples. These technical discrepancies may also partly explain differences in results between the two studies. Future clarifying studies should investigate blood plasma and a large number of samples. The study by Martelli-Palomino et al. showed that *UTR-1 (14 del/+3142C /+3187G)* is a high secretor, whereas *UTR-5* and *UTR-7 (14 ins/+3142G/+3187A)* are low secretors, while the rest of the haplotypes show intermediate sHLA-G levels [29]. *UTR-1* includes the *14 del* allele. This is in accordance with previous studies showing that the *14 ins * sequence and *+3142G* are associated with lower HLA-G expression [50, 65, 66]. The *14 del* allele is also found in *UTR-3*,* UTR-4*, and *UTR-6*, but all of these have the *+3187A* allele, whereas *UTR-1* displays the *+3187G* allele, which might also contribute to *UTR-1* showing higher sHLA-G expression than the other haplotypes. Of interest is also the fact that the *G∗01:05N* allele and the *G∗01:06* are derived from the *UTR-2* lineage, which was shown to be a low or intermediate secretor. This is in accordance with the study by Loisel et al. examining the *G∗01:05N* allele, as discussed above, and a study by Moreau et al. linking the *G∗01:06* fetal genotype to preeclampsia [45, 58]. In addition, the *UTR-2* is the only haplotype displaying a G at position +3196, whereas the other haplotypes display a C. From this it can be speculated that the *+3196G* allele might be a binding site for miRNA or the target of other regulatory mechanisms affecting HLA-G expression. No studies have linked this SNP to sHLA-G expression yet, and curiously no miRNA binding sites were found at positions +3187 and +3196 when testing with an array of miRNAs identified by affinity calculations, whereas binding sites were localized at positions +3003, +3010, +3027, +3035, and +3142 and at the 14 bp* *ins/del polymorphic site [61]. However, these two SNPs are located in close proximity to an AUUUA-pentamer sequence (Figure 1). Such AU-rich elements (AREs) have been described in the 3′UTR of labile mRNAs encoding, for example, cytokines. Therefore, sequence variation close to AREs may influence mRNA stability [67, 68].

At present, it seems that the *14 ins * polymorphism and the *+3142* SNP are the most important gene variations independently correlated with HLA-G protein expression. Also, a consensus can be made in the direction that classification based only on the *14 bp HLA-G* polymorphism will result in low-to-medium sHLA-G secretors in healthy donors with *14 ins/14 ins * genotypes and medium-to-high secretors for *14 del/14 del* genotypes.

## 3. Are There Any Functional Differences between Membrane-Bound and Soluble HLA-G?

Accumulating evidence suggests that the membrane-bound HLA-G1, the shedded sHLA-G1, the soluble HLA-G5, and possibly other isoforms might exhibit different functions during pregnancy. Early studies have not been able to support this because no antibodies have been available for distinguishing HLA-G isoforms. Given that shedded sHLA-G1 levels seem to be lower in blood plasma of pregnant women with severe preeclampsia in late pregnancy, as discussed earlier, it can be speculated that HLA-G1 might be the most important source of HLA-G in the pathogenesis of preeclampsia. However, as this effect is observed in late pregnancy, where the interface involving the VT and ST cells seems to play the most important role, the results are puzzling. HLA-G5 might be the most important isoform in the uterine compartment, although this is controversial. It might also be that the sHLA-G1 released during second trimester has important interactions with maternal peripheral immune cells, thereby inducing tolerance to the fetus. The role of circulating HLA-G5, though, still remains to be elucidated. A recent study based on HLA-G sequences transduced into K562 cells examining differences between the *14 ins* and the *14 del* alleles actually found that membrane-expression of HLA-G1 was higher in the *14 ins* transfectants than in the *14 del* transfectants. On the contrary, the 14* *del allele showed higher secretion rates of the shedded HLA-G1 than the *14 ins* allele. Furthermore, it was shown that the *14 ins* transfectants were more efficient in inhibiting NK cytotoxicity than the *14 del* transfectants in accordance with a high HLA-G1 expression [32].

In the male reproductive system, HLA-G5 seems to be the central molecule. Our group and Langat et al. have detected HLA-G5 in tissues such as the testis, the epididymis and the prostate gland, and sHLA-G expression in seminal plasma [16, 17]. Levels of sHLA-G in blastocyst media from *in vitro* fertilization (IVF) have also been examined, and it has been shown that high sHLA-G levels correlate well with fertility success [69, 70]. However, these studies did not differentiate between HLA-G isoforms and no studies have tried to correlate HLA-G expression in blastocysts and in IVF media to genetic variations. One study did, however, use an antibody stated to capture *β*2m-free HLA-G molecules (4H84) [71], but cross reactivity with other class I molecules has been reported using this mAb, questioning these results [72].

As described above, HLA-G has been found to interact with the immune receptors ILT-2, ILT-4, and KIR2DL4. ILT-2 is expressed on the surface of a wide variety of immune cells including NK cells, CD4^+^ and CD8^+^ T cells, B cells, macrophages, and monocytes, whereas ILT-4 is predominantly expressed on the surface of APCs such as macrophages, monocytes, and dendritic cells [22]. Another receptor for HLA-G is KIR2DL4, and up until now HLA-G has been thought to be the only known ligand for this receptor; however, this was recently challenged [73]. KIR2DL4 is mainly expressed on CD56^bright^ NK cells, the major proportion of NK cells in the uterus, whereas this cell type is almost exclusively absent in the pool of NK cells circulating in peripheral blood [35]. It was shown that membrane-bound HLA-G induced inhibition of uterine NK cell-mediated cytolysis through KIR2DL4, whereas peripheral NK cells were almost devoid of this receptor and conversely did not show inhibition of cytolysis [74, 75]. However, inhibition of peripheral NK cell cytotoxicity by HLA-G1 in an EVT cell line has been demonstrated. Additionally, KIR2DL4 surface expression was upregulated when cocultured with the HLA-G positive TEV1-cell line [76, 77]. Interestingly, KIR2DL4 has not yet been shown to interact with soluble HLA-G although a concept of endosomal signalling between these two has been suggested [78]. Intriguingly, a woman with several successful pregnancies has been identified, homozygous for a genotype not encoding KIR, stating that the interaction is not fundamental for successful pregnancy [79]. On the contrary, it has been shown that the expression of KIR2DL4 on the surface of uterine NK cells was higher in fertile women than among RSA women, indicating that the interaction between membrane-bound HLA-G and KIR2DL4 may favour induction of tolerance at the materno-fetal interface [80]. Moreover, shedded HLA-G has also been found to have the capacity to prevent NK-mediated cell lysis in a sHLA-G transfected HLA-negative cell line [81]. This study did not characterize any receptor interactions, making it difficult to determine whether the inhibitory effect is due to interaction with KIR2DL4, or alternatively another immune receptor such as ILT-2. One study indicated that HLA-G5 is more potent than HLA-G1 in inhibiting NK cell-mediated lysis, when HLA-G1 and -G5 transfectants were studied in K562 cells. However, the combination of HLA-G1 and -G5 had a significantly additive effect on the inhibition of NK cytotoxicity [82]. A recent study shows that the KIR2DL4 receptor also has the potential of acting through its activating motif. In a transfection study, it was shown that cytotoxicity of NK cells towards a cell line could actually be induced by the receptor interaction of KIR2DL4 and the unconventional *β*2m-free HLA-G isoforms. In addition proinflammatory cytokines such as IL-1*β*, TNF-*α*, and IFN-*γ* were expressed [83]. Since some studies point towards that *β*2m-free HLA-G isoforms are expressed at the fetomaternal interface, one could speculate that these proinflammatory cytokines participate in angiogenesis leading to vascular remodelling and migration of the trophoblast. This is in accordance with another study showing that sHLA-G does not affect the cytolytic activity of uterine mononuclear lymphocytes but induces IFN-*γ* secretion in both uterine and peripheral NK cells [84]. However, these findings are in direct contrast to the traditional view of pregnancy as an immunological shift from a Th1 to a Th2 response. Based on what we know today, this hypothesis seems to be too simplistic, and it is possible that membrane-bound HLA-G interacts with inhibitory immune receptors to induce tolerance of the fetus, and at the same time sHLA-G is serving as an activating molecule promoting proinflammatory cytokine secretion allowing trophoblast migration and vascular remodelling. It is hypothesized that the interaction between early trophoblast cells and endothelial cells of the spiral arteries is crucial for trophoblast invasion. This interaction has been shown to increase in a proinflammatory environment characterized by cytokines such as TNF-*α* and IL1*β* [85]. This study showed, by using blocking antibodies, that the adhesion molecules VCAM-1 and *α*4*β*1 were crucial for the interaction, but whether there is any interaction between these adhesion molecules and HLA-G on trophoblast cells remains to be elucidated. A recent study on JAR and JEG-3 cell lines found that HLA-G5 was able to stimulate trophoblast invasion through KIR2DL4 and ILT-2 probably through the ERK pathway [86]. This is in contrast to a previous study showing that sHLA-G actually inhibited trophoblast invasion [87]. Interestingly, these studies varied in concentrations of sHLA-G added in the invasion assay. In the study that suggested inhibition of trophoblast invasion, recombinant sHLA-G protein was added to the trophoblast cells at a concentration one hundred times higher in comparison to the study reporting a stimulation of trophoblast invasion. Taken together these studies could indicate that the effect of sHLA-G on trophoblast invasion was concentration-dependent.

In addition to the HLA-G receptor interactions described above several other immune cells might contribute to the induction of tolerance at the materno-fetal interface. Amodio et al. identified a specific dendritic cell population by flow cytometric analysis on first trimester decidual samples from healthy pregnancies undergoing elective abortions [88]. The DC-10 can either be recruited from peripheral blood, by induction of resident decidual dendritic cells, or by de novo induction promoted by the decidual microenvironment and have been shown to express high amounts of HLA-G and ILT-4 and promote IL-10 secretion. The IL-10 secretion is proposed to induce expression of HLA-G, ILT-2, and ILT-4 on immature decidual cells converting them to DC-10. The DC-10 cells can be important in inducing tolerance as they have been shown to be potent inducers of a specific subset of CD4^+^CD25^+^FOXP3 regulatory T cells called Tr1 cells *in vitro* [89]. Another specific subset of regulatory CD4^+^ T cells constitutively expressing HLA-G has been shown to accumulate at inflammatory sites [90]. The study by Amodio et al. also showed that levels of CD4^+^HLA-G^+^ T cells were significantly higher in the peripheral blood of pregnant women compared to healthy controls [88]. From this it can be speculated that these cells are recruited to the fetomaternal interface during early implantation, where inflammatory responses might be involved in trophoblast invasion. In addition, these HLA-G positive cells have been shown to suppress T-cell proliferation through a reversible regulation of inflammation dependent on IL-10 and HLA-G [91].

## 4. Tissue Specific HLA-G Expression in relation to *HLA-G* Genetics

At present, not many published studies have addressed possible differences in *HLA-G* genotype-associated expression between different types of cells and tissues. This could be accomplished by cell- or organ-specific differences in stimulatory or inhibitory substances, for example, hormones, or for independent types of cells, transcription factors or miRNAs. A range of studies have been published that show significant associations between sHLA-G concentrations in blood plasma and serum and the *HLA-G 14 bp ins/del* genotype, alternatively *HLA-G* 3′UTR haplotypes, as described previously [29, 50, 63, 65]. In these studies, homozygous *14 ins/14 ins * individuals show general lower sHLA-G1/HLA-G5 protein levels than *14 del/14 del* individuals, as discussed above. However, recently, we have shown that the *14 ins* allele has the highest membrane-bound expression of HLA-G1 in transduced K562 cells [32]. Altogether, the relationship between *HLA-G* genetics and HLA-G expression levels may turn out to be more complicated than previous thought, and it may even be tissue-specific.

## 5. Clarification of *HLA-G* Allele Associations in Preeclampsia and Related HLA-G Expression Is Needed

An increasing number of studies have indicated a role for HLA-G in the pathogenesis of preeclampsia. Several studies have reported reduced sHLA-G concentrations in maternal blood in preeclamptic cases compared to controls in all three trimesters of pregnancy [53, 71, 92–94]. HLA-G protein and mRNA expression in the placenta seem to be reduced in preeclampsia [40, 43, 44]. Furthermore, several studies have observed significant associations between certain *HLA-G* alleles, genotypes, and haplotypes [25, 38, 41, 45, 46]. Special attention has been drawn to the *14 ins* allele and an increased risk of severe preeclampsia in pregnancies, where the fetus is homozygous for an *HLA-G* 3′UTR haplotype that includes the *14 ins*, *+3010C*, *+3142G*, *+3187A*, and *+3196G *polymorphisms [25]. Whether this 3′UTR haplotype is a low or intermediate sHLA-G secretor in healthy donors is currently controversial [29, 63]. Furthermore, it is not known if these findings can be extrapolated to HLA-G expression in trophoblast cells, and thereby maternal blood sHLA-G levels during pregnancy, which is higher than in nonpregnant women. The soluble HLA-G concentration during pregnancy must be a mix of contributions from the mother, most probably from maternal immune cells, and from the fetal trophoblast cells in the placenta. Two studies suggest that the relationship between *HLA-G* polymorphism and HLA-G expression during pregnancy might be complex. A small study of HLA-G expression in term placenta in relation to *HLA-G* genotypes and polymorphisms using immunohistochemical staining of HLA-G indicates that *14 ins/14 ins * trophoblast cells do not show a clearly reduced expression of HLA-G [95]. Finally, a recent study of *14 ins* and *14 del* transfectants in the K562 cell line revealed that the *14 ins *transfectants had a higher cell surface expression of HLA-G1 than the *14 del* transfectants [32]. These controversies need to be clarified in future studies. In relation to a possible importance of HLA-G expression in the pathogenesis of preeclampsia, it is important to study the influence of the *HLA-G* polymorphisms in the 5′URR and the 3′UTR on transcription, mRNA stability, and alternative splicing. This is also important in the context that there might be differences between HLA-G expression linked to these polymorphisms in trophoblast cells and in immune cells. It can be speculated that this might be due to different profiles of miRNAs, other regulatory factors, and methylation status. Some of these interactions might be abnormal in preeclampsia and associated with specific *HLA-G* haplotypes, for example, the *14 ins/+3142G/+3187A* haplotype as one study indicates [25].

Maybe the predominating soluble HLA-G isoform in nonpregnant female donors and in male donors is HLA-G5, and during pregnancy the rise in sHLA-G in the maternal blood might primarily be a result of shedded HLA-G1 from trophoblast cell membranes in the placenta. Therefore, HLA-G protein expression in trophoblast cells in relation to *HLA-G* genetics needs to be investigated in more detail.

That the reduced sHLA-G blood levels observed in preeclampsia by a range of studies should merely be a result of a specific fetomaternal *HLA-G* genotype combination is probably not the case. It can be hypothesized that it might be a combination of predisposing *HLA-G* polymorphism in interaction with one or several other possible pathogenic factors, for example, an aberrant miRNA profile, defects in metalloproteinase activity that have been reported in preeclampsia, or the presence of certain viruses in the placenta that contribute to development of preeclampsia [96, 97]. Several studies have elucidated how human cytomegalovirus (HCMV) interferes with and downregulates HLA-G expression [98, 99]. Interestingly, a small pilot study has linked the presence of HCMV sequences and certain *HLA-G* alleles with increased risk of preeclampsia, and there might be some evidence for an association between CMV infection and preeclampsia [100, 101].

In conclusion, in future studies for clarification of the role of HLA-G in the development of preeclampsia, ideally *HLA-G* genetics, maternal blood sHLA-G levels, metalloproteinase activity, and the presence of specific viruses should be studied in the same cohort of pregnant women including a substantial number of pregnancies complicated with preeclampsia.

## Figures and Tables

**Table 1:** A comparison of differences in *HLA-G* gene and protein expression and functional characteristics between the *14 bp deletion* and *14 bp insertion* alleles. The observed differences may be influenced by other DNA and mRNA polymorphisms in the 3′-untranslated region (3′UTR), especially the *+3142* and *+3187* SNPs, and/or the 5′-upstream regulatory region of HLA-G that are in linkage disequilibrium with the *14 bp ins/del* polymorphism (see the text and Figure 1 for details). (Based on a large number of references listed in the text).

	*14 bp * *deletion * *HLA-G* allele	*14 bp * *insertion * *HLA-G* allele
Alternative splicing of *HLA-G* mRNA that includes a deletion of 92 bp of the 3′UTR	No	Yes

Levels of *HLA-G* mRNA (not including the 92 bp splice variants)	+++	++

Levels of soluble HLA-G1	?	?

Levels of HLA-G5 during pregnancy*	+	++

Levels of soluble HLA-G in blood plasma from healthy nonpregnant donors**	+++	++

Membrane-bound expression of HLA-G1*	++	+++

*HLA-G* mRNA stability	+	+++

Inhibition of NK cytotoxicity*	+	++

*Only one or very few studies; needs further verification.

**β2-microglobulin-associated soluble HLA-G1 and HLA-G5.
